# Model Systems for Addressing Mechanism of Arrhythmogenesis in Cardiac Repair

**DOI:** 10.1007/s11886-021-01498-z

**Published:** 2021-05-29

**Authors:** Xiao-Dong Zhang, Phung N. Thai, Deborah K. Lieu, Nipavan Chiamvimonvat

**Affiliations:** 1 Division of Cardiovascular Medicine, Department of Internal Medicine, School of Medicine, University of California, Davis, Davis, CA 95616 USA; 2 Department of Veterans Affairs, Veterans Affairs Northern California Health Care System, Mather, CA 95655 USA; 3 Department of Pharmacology, School of Medicine, University of California, Davis, Davis, CA 95616 USA

**Keywords:** Cardiac arrhythmias, Cardiac cell-based therapy, Human-induced pluripotent stem cells, Animal models of cardiac arrhythmias, Patient-specific disease models, Genome editing, Cardiac hypertrophy, Heart failure

## Abstract

**Purpose of Review:**

Cardiac cell-based therapy represents a promising approach for cardiac repair. However, one of the main challenges is cardiac arrhythmias associated with stem cell transplantation. The current review summarizes the recent progress in model systems for addressing mechanisms of arrhythmogenesis in cardiac repair.

**Recent Findings:**

Animal models have been extensively developed for mechanistic studies of cardiac arrhythmogenesis. Advances in human induced pluripotent stem cells (hiPSCs), patient-specific disease models, tissue engineering, and gene editing have greatly enhanced our ability to probe the mechanistic bases of cardiac arrhythmias. Additionally, recent development in multiscale computational studies and machine learning provides yet another powerful tool to quantitatively decipher the mechanisms of cardiac arrhythmias.

**Summary:**

Advancing efforts towards the integrations of experimental and computational studies are critical to gain insights into novel mitigation strategies for cardiac arrhythmias in cell-based therapy.

## Introduction

Cardiovascular disease is the leading cause of morbidity and mortality worldwide and causes more deaths than all cancers combined [1]. Despite significant advances in therapy and management, heart failure (HF) remains a life-threatening disease with a 5-year mortality rate of 45–60% [1]. Therefore, there is a compelling need to seek new options for patients suffering from HF. Since adult cardiac myocytes are unable to proliferate sufficiently to replace the damaged tissue, stem cell therapy represents a promising approach for the treatment of end-stage HF, since it aims at generating new functional myocardium and inducing neoangiogenesis. However, therapeutic strategies using cell-based therapy have not produced full restorative functions [2, 3]. A high rate of transplanted stem cell loss (90% within the first few days) has been observed [4, 5]. Moreover, stem cell transplantation has been shown to be associated with occurences of cardiac arrhythmias [6•, 7••, 8, 9], which represents one of the main challenges in the field of cardiac cell-based therapy.

Animal models have been extensively developed for mechanistic studies of cardiac arrhythmogenesis [10•, 11–13], enabling genetic modification using gain- and loss-of-function strategies. However, limitations exist including significant electrophysiological differences between human and animal hearts, costs, as well as ethical considerations. To circumvent some of these shortcomings, cellular and multicellular models for arrhythmogenesis have been widely used, which are further enhanced by advances in human-induced pluripotent stem cells (hiPSCs), tissue engineering [14–19], and gene editing. The hiPSC-derived cardiomyocytes (hiPSC-CMs) can be obtained from healthy or diseased individuals, and provide the inexhaustible source for human disease modeling. Finally, recent development in multiscale computational studies provides yet another powerful tool to quantitatively test the mechanisms of cardiac arrhythmias in cardiac repair [20••].

The current review will summarize the recent progress in model systems for addressing mechanisms of cardiac arrhythmogenesis, and serve as a discussion platform to gain insights into mechanistic underpinnings and novel mitigation strategies for arrhythmogenesis in cardiac repair.

## Animal Models for Cardiac Arrhythmias

**Fig. 1:**
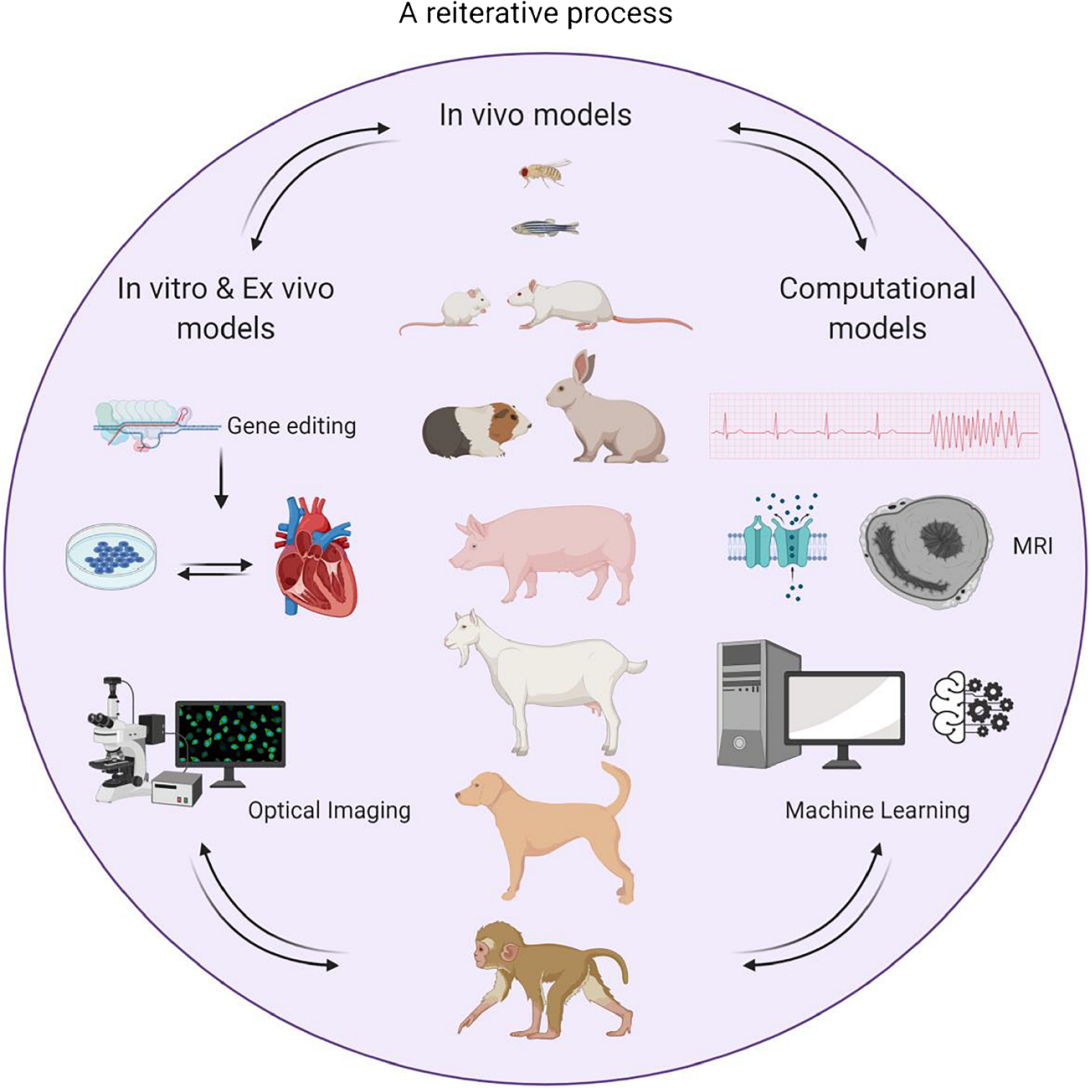
Summary of the model systems for investigating the mechanism of arrhythmogenesis in cardiac repair demonstrating the reiterative process for the research strategies (figure generated using BioRender)

Numerous species have been utilized to study the underlying mechanisms of arrhythmogenesis, ranging from small animals such as zebrafish to large animal models such as pigs and nonhuman primates (Fig. 1). Although no animal models perfectly replicate arrhythmogenesis seen in humans, the multitudes of animal models have substantially advanced our understanding of different aspects of arrhythmogenesis. Small animal models are generally used for mechanistic discoveries, drug screening, and testing, while larger animals are reserved for validation of these key findings and to further establish drug safety profiles.

### Cardiac Hypertrophy

Electrical and structural remodeling in pathological cardiac hypertrophy has been shown to increase patient’s susceptibility to ventricular arrhythmias (VAs) and sudden cardiac death (SCD). Electrical remodeling in cardiac hypertrophy is well documented and includes action potential (AP) prolongation [21] and conduction delay [22]. One of the most commonly used animal models of cardiac hypertrophy is surgical aortic constriction, which elevates afterload and consequently induces adverse structural and electrical remodeling. Aortic constriction is commonly used in mouse models due to the ease of genetic manipulation; however, it has been adapted to rats [21], guinea pigs [23], rabbits [24], and pigs [25]. Additionally, animal models with volume overload can induce pathological remodeling of the heart that result in cardiac hypertrophy, such as arterio-venous shunt formation and aortic regurgitation [26].

### Myocardial Ischemia and Infarction

Coronary artery disease (CAD), leading to myocardial ischemia and infarction, is a frequent cause of cardiac arrhythmia. Cardiac ischemia and infarction result in complex electrophysiological remodeling [27•]. In murine models, myocardial infarction is typically induced by ligation of the left anterior descending (LAD) coronary artery. In larger animal models, ligation of other coronary arteries such as the left circumflex or right coronary artery has also been used [28].

### Atrial and Ventricular Tachyarrhythmia

Atrial fibrillation (AF) is the most prevalent cardiac arrhythmia seen clinically [29]. A commonly used model of AF is atrial tachypacing. Although it is employed in dogs [30], sheep [31], pigs [32], and rabbits [33], its usage has not been extended over to smaller animals due to their significantly higher heart rates [34]. Tachypacing may also be used on the ventricles to study HF-induced arrhythmogenesis [35].

### Heart Failure

Heart failure (HF) occurs when the cardiac output is no longer able to meet the metabolic demands of the body, and is frequently associated with cardiac arrhythmias [36]. Animal models of cardiac hypertrophy, myocardial ischemia/infarction, and atrial and ventricular tachypacing [35] can produce HF when the intervention is prolonged, combined, and/or severe. Although not necessary in smaller animals, larger animals may require more than one intervention to induce HF. For instance, a commonly used rabbit model of HF involves aortic valve cusp perforation, followed by aortic constriction, to create a volume and subsequent pressure overload [26]. These multiple interventions are required since HF is uncommon with pressure overload in rabbits, but they may induce an increase in mortality.

### Genetic Models

Gene-targeted animal models predominantly in mice have greatly expanded our mechanistic understanding of long QT syndrome (LQTS), short QT syndrome (SQTS), Brugada syndrome, catecholaminergic polymorphic ventricular tachyarrhythmia (CPVT), sick sinus syndrome, cardiac conduction disease, and familial AF [37]. Genetic manipulation in larger animals have also been described including LQTS 1 and 2 models in rabbits, overexpression of *TGFB1* in goats, and mutated *SCN5A* in pigs [10•]. Indeed, emerging technologies for genome editing, including clustered regularly interspaced short palindromic repeats (CRISPR)-Cas9 mediated gene editing, have enabled the generation of genetic models for arrhythmias in larger animals.

Animal models have significantly expanded our understanding of the mechanistic underpinning of arrhythmogenesis, but there exist advantages and limitations that should be considered. Besides the obvious issues with costs and the length of time required for model generation, there are inherent species-dependent differences at the molecular and cellular levels that impact pathophysiology. Indeed, these differences must be evaluated and understood in order to translate therapeutic findings to clinical practice. Advantages of using small animal models are their relatively inexpensive costs, short gestational period and large litter size, and relative ease of genetic manipulation. However, small animals possess cardiovascular anatomy and physiology that are substantially different from humans [34]. For instance, AP profiles, electrocardiograms, and heart rate are significantly different between humans and rodents [10•]. These differences make larger animal models more suitable for cardiac electrophysiology and arrhythmia studies. Nonetheless, the usage of small animal models in arrhythmia research provides valuable insights, guides the design of studies in larger animals, and eventual translation to humans.

## Ex Vivo Models for Cardiac Arrhythmias

Studies in cardiac arrhythmogenesis have also taken advantage of the ex vivo Langendorff-perfused heart (Fig. 1). Isolated hearts from rabbits, guinea pigs, sheep, and mice have been extensively used to investigate cardiac repolarization [38, 39], AF [40, 41], ventricular fibrillation (VF) [42–44], torsades de pointes (TdP) [45], AV node conduction [46, 47], and hypokalemia-induced arrhythmia [38, 48, 49]. Explanted human hearts from patients who undergo heart transplantation have been invaluable in cardiac arrhythmia studies [44]. Additionally, ex vivo Langendorff-perfused hearts are employed for drug testing [46, 50–54]. The ex vivo studies enable regional-specific interrogations including cardiac conduction systems, sinoatrial and atrioventricular nodes, regional heterogeneity, and cell-cell interactions. Finally, advanced imaging techniques such as optical or electrical mapping, two-photon microscopy, and optogenetics have greatly enhanced the impact of these ex vivo models [55–60]. One main pitfall of the model is the absence of cardiac innervation, which is a critical factor for arrhythmogenesis.

## Cellular and Tissue Models for Arrhythmogenesis

For deciphering the mechanisms of arrhythmogenesis, in vitro models are useful tools that can simplify experiments by restricting the factors of interest to be tested independently. The models vary from murine to human cardiomyocytes and from single cells to multi-cellular preparations (Fig. 1). Each of these models is associated with distinct advantages and disadvantages.

### Cardiomyocytes From Animal Models

Primary cardiomyocytes can be harvested from animal models and are critical for our understanding of mechanisms of arrhythmias at the cellular and subcellular levels. The source of focal arrhythmic activity—spontaneously generated or triggered AP—either due to ion channelopathies, altered ion channel expression, subcellular localization, or post-translational modifications, and drug-modulated channel function, can be readily deciphered using patch-clamp and imaging techniques. Myocardial infarction models exhibiting arrhythmias in vivo that have been attributed to reduced Ca^2+^ or K^+^ currents were determined in single cardiomyocytes [61, 62]. Underlying cellular mechanisms of arrhythmias in atrial and ventricular tachycardia models were similarly determined in isolated single cells [63]. Studies using single cardiomyocytes enable focused experiments on the cellular and subcellular mechanisms in the absence of other contributing pro-arrhythmic factors, such as fibrosis.

### Cell Lines of Cardiomyocytes

A readily available source of cardiomyocytes is highly desirable for in vitro studies; however, the lack of regenerative capability of adult cardiomyocytes requires that primary culture is used as the main source. To circumvent limited proliferative potential of cardiomyocytes, cell lines have been generated by restoring the proliferative ability. This can be achieved by infecting cardiomyocytes with simian virus (SV) 40, a type of DNA tumor virus, that expresses large and small T antigens, which cooperatively inhibit tumor suppressors and transform the cells to escape senescence [64]. HL-1 cells are commercially available, immortalized murine atrial cardiomyocytes that originated from a subcutaneously engrafted atrial tumor, AT-1 expressing SV40 large T-antigen under the atrial natriuretic factor promoter, in C57BL/6J mice [65]. The ability of this cell line to expand indefinitely in vitro and to allow cryo-storage, while maintaining the characteristics and function of cardiomyocytes, make this cell line an attractive alternative to primary cardiomyocytes. Since its derivation, this line has been used in a large number of studies, including those investigating reentry spiral waves [66]. A human cardiomyocyte line, AC16, has been similarly generated through fusion of primary ventricular cardiomyocytes with a SV40-immortalized human fibroblasts [67]. Although this cell line expresses cardiomyocyte-specific markers and exhibits outward currents, it is unable to generate APs due to its deficiency in inward currents. This caveat in electrophysiological characteristic makes it unsuitable for arrhythmia studies.

### HiPSC-Derived Cardiomyocytes

Considerable insights in cardiac arrhythmias have been derived from various species. However, the mechanisms responsible for arrhythmogenesis in the context of human electrophysiology may be significantly different from animal models with differing electrophysiology. Therefore, human cardiomyocytes remain the most reliable model for investigating the mechanisms of human arrhythmias. Primary human cardiomyocytes are not easily obtainable. Their laboratory usage is further limited by the difficulty of maintenance in culture. HiPSCs that can differentiate into all somatic cell types, including cardiomyocytes, have garnered great interest in recent years as a readily available option. These cells not only open the possibility of having an unlimited cell source as well as genetic modification or imposed environmental conditions but hiPSCs can also be generated from patients suffering from cardiac arrhythmias to generate patient-specific in vitro models. To date, numerous LQTS-, SQTS-, Brugada syndrome-, and CPVT-hiPSC lines have been generated to study the channelopathies [68–73]. Cardiomyocytes differentiated from these diseased lines faithfully exhibit abnormal APs and arrhythmias as expected. The predictive value of hiPSC-derived cardiomyocytes for drug testing and safety screening has also been demonstrated [74–76]. Additionally, this model has been utilized to iteratively improve the moiety of a target compound, mexiletine, for therapeutic potency and safety [77].

HiPSC-derived cardiomyocytes are not, however, without disadvantages. There are two key issues associated with using these cells for studying arrhythmias: (1) the heterogeneity of cardiomyocyte subtypes in the differentiated population and (2) immaturities of hiPSC-derived cardiomyocytes. Widely used cardiomyogenesis protocols to differentiate hiPSCs yield a heterogeneous population of pacemaker-, atrial-, and ventricular-like subtypes, as classified by the AP profiles [78]. The heterogeneity in electrophysiology among the subtypes can mask dysfunctional effects due to differential expression of ionic currents. Indeed, only the atrial and ventricular subtypes differentiated from LQT1-patient hiPSCs exhibit significantly prolonged AP duration, while the electrophysiology of the pacemaking subtype appears normal [79]. The results highlight the importance of using the appropriate subtype for studying the arrhythmia in question.

A few strategies have been devised to minimize subtype impurities. Enrichment of desired cardiomyocyte subtype requires a surface marker with high specificity for the subtype. Ventricular cardiomyocyte enrichment by selecting CORIN-positive or CD77-positive/CD200-negative cardiomyocytes has been reported, but the specificity could still be improved [80, 81]. An alternative strategy to reduce undesired cardiomyocyte subtype is direct differentiation to a specific subtype. Directed differentiation protocols that promote a higher fraction of ventricular, atrial, or pacemaking subtype have been reported [82–88]; however, none of the protocols could attain 100% yield of the desired subtype.

Immaturity of hiPSC-derived cardiomyocytes compared to adult cells, from excitation to contraction, is another issue that may confound experimental results. For instance, the pacemaking, hyperpolarization-activated cyclic nucleotide-gated (HCN4) channels that are absent in the adult working cardiomyocytes are present in all hiPSC-derived cardiomyocytes [89]. Conversely, inwardly rectifying Kir2.1 channels that are present to stabilize the membrane potential in adult working cardiomyocytes are largely absent in hiPSC-derived cardiomyocytes [90]. Consequently, all hiPSC-derived cardiomyocytes exhibit automaticity or phase-4 depolarization, both of which can be a trigger for arrhythmia. Therefore, only a preparation with stable baseline response should be used for the experiments. Whenever possible, the baseline response should also be recorded for comparison against induced dysfunction. The lack of Kir2.1 also means that cardiomyocytes differentiated from a LQT7 patient-specific hiPSC line are unlikely to be good candidates to study this particular channelopathy until these cells further mature.

To remedy this developmental deficiency, maturation strategies have been devised, which range from electrical, mechanical, to metabolic conditioning [90–95]. Significant improvements in all functional aspects have been observed with maturation conditioning, but none can attain the characteristics matching the adult cells. Despite immaturities, hiPSC-CMs have been used to study numerous channelopathies and for drug screening with expected presentation of dysfunction and accuracy [68–76]. Of note, immature hiPSC-derived cardiomyocytes have been reported to exhibit greater sensitivity to pro-arrhythmogenic drugs than those that have undergone the maturation process. Increased rate of false-positive arrhythmic events needs to be taken into account when using these cells for drug screening.

### Tissue Models

One arrhythmogenic mechanism that fails to be captured at the single cell level is the impulse propagation in arrhythmia formation. Ectopic beats triggered by automaticity, early or delayed after depolarizations (EADs or DADs) are focal events that only lead to arrhythmia if these are propagated to the neighboring cells. Consequently, arrhythmias can only be observed in multi-cellular tissue models. Besides cellular contribution of abnormal electrophysiology, cell-cell electrical coupling affecting the conduction velocity is another factor that dictates the wave length of the AP wave front. Additionally, electrophysiological heterogeneities, including dispersion of repolarization or source-sink mismatch, are triggers for arrhythmias that only become apparent at the tissue levels.

Conduction velocity and wave front reentry can be observed in either 2D or 3D tissue models composed of a syncytium of cardiomyocytes via optical mapping. Since primary adult cardiomyocytes from animals do not form monolayers in culture, most in vitro tissue models have relied on primary neonatal or hiPSC-derived cardiomyocytes that retain morphological plasticity to reorganize and allow electrical coupling with neighboring cells. For the 2D model, cardiomyocytes plated in typical cell cultureware exhibit random cellular orientation unlike the anisotropic alignment of the myocardium. Conduction pattern is affected by the cardiomyocyte alignment, which is reflected in the increased incidence of arrhythmic events even in healthy monolayer of hiPSC-derived cardiomyocytes, making this model less accurate for testing pro-arrhythmic triggers. With advances in microfabrication technology, patterned surface to induce aligned monolayers of hiPSC-derived cardiomyocytes has been shown to reduce the incidence of arrhythmia, creating a more stable baseline tissue model for assessing pro-arrhythmic factors [96, 97].

While 2D hiPSC-cardiomyocyte-based tissue models can display arrhythmic events, there are limitations in the organization and maturity of cardiomyocytes. Specifically, 3D tissues in a patch configuration have been reported to be necessary in modeling arrhythmic phenomenon such as torsade de pointes with fluctuating excitation intervals that can only be recreated in the presence of a meandering wave origin [98]. This is not possible in a 2D configuration with a stationary wave center. Other 3D hiPSC-derived cardiomyocyte-based tissue configurations for testing arrhythmic events include linear or circular strips, small microtissues, and organoid chambers [16, 99–103]. These tissue models present a more mature cardiomyocyte phenotype than the 2D counterpart. However, most 3D cardiac tissues in the literature have added fibroblasts to facilitate tissue compaction. Depending on the fibroblast type, the electrical coupling with the cardiomyocytes may differ, which may result in different source-sink ratio that can affect the conduction pattern and intrinsic arrhythmogenicity [104]. Therefore, there are many factors to consider when choosing a tissue model for in vitro arrhythmia studies.

## Mathematical Models for Addressing the Mechanisms of Arrhythmogenesis

Since cardiac excitability is sculpted by the beat-to-beat feedback among three nonlinear dynamic processes including Ca^2+^ dynamic, APs, and mechanical contractions, multiscale computational modeling offers unparalleled advantages in deciphering cardiac arrhythmia mechanisms (Fig. 1). The fundamental question is how to fully integrate experimental and clinical information and mathematical modeling to accurately simulate cardiac structural and electrophysiological properties at subcellular, cellular, tissue, and organ levels. This multiscale modeling is a challenge considering the complexity and heterogeneity of cardiac tissues. With the recent development of computational techniques, research in cardiac computational modeling has greatly expanded with the support from experimental and clinical investigators [105–109]. Computational models of different cell types including sinoatrial node (SAN) cells, atrial myocytes, atrioventricular (AV) node cells, Purkinje fibers, and ventricular myocytes have been developed. For understanding human cardiac diseases, many computational models across species have been developed using experimental data from animal studies for validation and comparison. Here, we will focus on the human cardiac models.

### Atrial and AV Node Models

The early mathematical models of the AP from adult human atrial cells were developed by Nygren et al. and Courtemanche et al. in 1998 [110, 111]. With accumulation of human atrial experimental data, several human atrial models have been refined including the AV node [112–120]. The significance of these models was demonstrated by simulating the AP alternans, AF, conduction block, and AF-associated electrical remodeling in single cells, one-, two-, and three-dimensional tissues [112, 116, 121–124]. Computational models are powerful tools to study the mechanism of AF, and have the potential to guide clinical treatment of AF using catheter ablation [125, 126]. In the past decade, 3D computational modeling has been used to guide and optimize AF ablation and therapy to minimize the ablation lesions and improve clinical outcomes [107].

### Ventricle Models

The first mathematical model of the AP from human ventricular myocytes was reported by Priebe and Beuckelmann in 1998 [106, 127]. The models for ventricular myocytes and tissues were further refined using larger human dataset [106, 128–133]. The models have been used to explore and predict the mechanism of initiation, maintenance, and termination of ventricular arrhythmias, including simulations of triggered activities, AP and conduction velocity restitutions, inherited arrhythmias, prediction of AP alternans, the vulnerable window, and efficacy of antiarrhythmic drugs [106]. More recently, computational models have been developed and applied to characterize ventricular arrhythmias under different clinical settings and diseased states including heart failure, cardiac ischemia, and maintenance of torsades de pointes [107, 134–138]. Multiscale modeling has been successfully used to demonstrate the origins of ECG morphology and abnormal ECG features resulted from diseases [139–143]. With the aid of the advanced computational techniques, modeling of patient-specific cardiac diseases becomes feasible with successful applications in predicting patient’s risk for ventricular arrhythmias and SCD [144, 145]. Treatment of ventricular tachycardia and defibrillator implantation have also been facilitated by using computational modeling to predict the optimal ablation targets and defibrillation locations [146, 147].

### Purkinje Fiber Models

Purkinje fibers play critical roles as initiating sites for ventricular tachycardia and fibrillation. An AP model of human Purkinje fibers was first developed by Tusscher and Panfilov [148]. Based on detailed biophysical kinetic analysis of the ion channels in human Purkinje fibers, a new computational model was proposed and applied to simulate LQTS [149, 150]. The 3D Purkinje fiber network model was established based on imaging data, which integrates the Purkinje fibers with the ventricle to simulate the electrical activation sequences in the ventricle [151]. Recently, ionic currents of human Purkinje-related electrophysiology, pacemaker activity, and arrhythmogenicity were further revealed by incorporating Purkinje-specific ionic currents and Ca^2+^ handling [152]. The structural and functional integration of ventricular and Purkinje fiber models will significantly improve our understandings of the mechanisms of ventricular arrhythmias. Additionally, the structural and functional contribution of cardiac fibroblasts also needs to be considered [153].

### SAN Models

Because of the very limited experimental data from human SAN, the development of mathematical model for human SAN lags behind the model development for human atrial and ventricular myocytes. Seemann et al. published the first human SAN AP model as part of a 3D human atria model [154]. This is followed by an AP model based on the mRNA expression of ion channels in healthy adult human SAN tissues [155]. A recent study integrates the membrane and Ca^2+^ clock and constructs a comprehensive mathematical model for SAN cells [156].

### Virtual Heart Models

One major advantage of computational modeling is the expansion of personalized medicine by integrating patient information and clinical data into the model to develop personalized treatment strategies [157]. A recent virtual heart technology for guiding the ablation of ventricular tachycardia demonstrates the highly promising future for treatment of cardiac arrhythmias [158]. The incorporation of machine learning and artificial intelligence in the diagnosis and treatment of cardiac arrhythmias will provide powerful tool sets for the prevention, prediction, and treatment of cardiac arrhythmias (Fig. 1) [159]. Indeed, multiscale 3D whole-heart modeling has recently been developed to determine how varying parameters of cell delivery and transdifferentiation could result in focal ectopy, heart block, and reentry [20••].

Finally, the innervation, spatial, and temporal dynamic feedback at tissue and organ levels are required to be integrated into the models for understanding the beat-to-beat electrical excitability in the heart. Therefore, joint efforts of computational biologists, experimental biologists, and physicians are necessary to integrate the multi-discipline knowledge in computational modeling. Additional validation with experimental and clinical data followed by reiterative refinement of the computational models will help to pave the way for deeper understandings of the complex mechanisms of cardiac arrhythmogenesis, assist the diagnosis, and guide the treatment.

## Conclusions and Perspectives

The mechanistic understanding of cardiac arrhythmogenesis is critical for the development of cardiac cell-based therapy. Animal models from multiple species provide experimental platforms for mechanistic hypothesis testing. Ex vivo and in vitro models enable the application of several cutting-edge techniques including the development of patient-specific hiPSC-CMs and CRISPR-Cas9 gene editing. Recent advances in multiscale computational models for arrhythmogenesis represent the joint efforts by computational biologists, biophysicists, experimental biologists, and physicians, and provide quantitative and powerful tools for the mechanistic understanding of arrhythmogenesis in cardiac cell-based therapy. Further integrations of experimental, computational, and the virtual heart model will provide critical insights into novel mitigation strategies for cardiac arrhythmias in cell-based therapy.
